# Unhealthy Diets Induce Distinct and Regional Effects on Intestinal Inflammatory Signalling Pathways and Long-Lasting Metabolic Dysfunction in Rats

**DOI:** 10.3390/ijms231810984

**Published:** 2022-09-19

**Authors:** Sofia Nogueira, Joana Barbosa, Juliana Faria, Susana I. Sá, Armando Cardoso, Raquel Soares, Bruno M. Fonseca, Sandra Leal

**Affiliations:** 1 TOXRUN—Toxicology Research Unit, University Institute of Health Sciences, University Institute of Health Sciences, Advanced Polytechnic and University Cooperative (CESPU), CRL, 4585-116 Gandra, Portugal; 2 UCIBIO, REQUIMTE—Applied Molecular Biosciences Unit, Laboratory of Toxicology, Department of Biological Sciences, Faculty of Pharmacy, University of Porto, 4050-313 Porto, Portugal; 3 CINTESIS@RISE, MEDCIDS, Faculty of Medicine, University of Porto, Rua Dr. Plácido da Costa, 4200-450 Porto, Portugal; 4 Unit of Anatomy, Department of Biomedicine, Faculty of Medicine of University of Porto, Al Prof Hernâni Monteiro, 4200-319 Porto, Portugal; 5 Unit of Biochemistry, Department of Biomedicine, Faculty of Medicine, University of Porto, Al Prof Hernâni Monteiro, 4200-319 Porto, Portugal; 6 i3S, Instituto de Investigação e Inovação em Saúde, Universidade do Porto, R. Alfredo Allen 208, 4200-135 Porto, Portugal; 7 Associate Laboratory i4HB—Institute for Health and Bioeconomy, Laboratory of Biochemistry, Department of Biological Sciences, Faculty of Pharmacy, University of Porto, 4050-313 Porto, Portugal; 8 UCIBIO, REQUIMTE—Applied Molecular Biosciences Unit, Laboratory of Biochemistry, Department of Biological Sciences, Faculty of Pharmacy, University of Porto, 4050-313 Porto, Portugal

**Keywords:** western diet, high-sugar diet, toll-like receptors, iNOS, intestinal inflammation, long-lasting metabolic effects

## Abstract

The intestinal epithelium is a principal site for environmental agents’ detection. Several inflammation- and stress-related signalling pathways have been identified as key players in these processes. However, it is still unclear how the chronic intake of inadequate nutrients triggers inflammatory signalling pathways in different intestinal regions. We aimed to evaluate the impact of unhealthy dietary patterns, starting at a younger age, and the association with metabolic dysfunction, intestinal inflammatory response, and obesity in adulthood. A rat model was used to evaluate the effects of the consumption of sugary beverages (HSD) and a Western diet (WD), composed of ultra-processed foods. Both diets showed a positive correlation with adiposity index, but a positive correlation was found between the HSD diet and the levels of blood glucose and triglycerides, whereas the WD diet correlated positively with triglyceride levels. Moreover, a distinct inflammatory response was associated with either the WD or HSD diets. The WD induced an increase in *TLR2*, *TLR4*, and nuclear factor-kappa B (*NF-κB*) intestinal gene expression, with higher levels in the colon and overexpression of the inducible nitric oxide synthase. In turn, the HSD diet induced activation of the *TLR2*-mediated *NF-κB* signalling pathway in the small intestine. Altogether, these findings support the concept that early intake of unhealthy foods and nutrients are a main exogenous signal for disturbances of intestinal immune mechanisms and in a region-specific manner, ultimately leading to obesity-related disorders in later life.

## 1. Introduction

Consumption of ultra-processed foods and sugary beverages are some of the main contributors to the development of obesity and its related comorbidities [1,2,3]. Although obesity is defined as abnormal body fat accumulation, the biological processes involved are complex and go beyond excessive energy intake. It is recognized that the activation of the immune system and chronic low-grade inflammation are mechanisms involved in obesity-related metabolic disease [4,5]. The disruption of the microbiota–host interactions for diet early in life may account for the deviant programming of immunity and metabolic disorders later in life [6,7,8,9]. Extensive research has suggested that the link between diet and obesity-induced inflammation seems to be closely related with an impaired intestinal barrier function [10,11,12,13,14,15], which can play a crucial role in the disruption of energy homeostasis. However, how macronutrients’ intake affects metabolic and immune systems is still uncertain. Moreover, the mechanisms underlying the link between obesity-related diets and metabolic inflammation remain poorly understood and may vary depending on tissue types and location.

The intestinal epithelium represents the largest interface for the occurrence of an intricate microbiota–host crosstalk, which is strongly influenced by luminal environment and internal signals [16,17,18]. Among the external cues, both dietary macronutrients and microbiota compounds interact with the intestinal barrier to modulate the signalling pathways controlling physiologic functions, such as digestion, absorption, immunity, and metabolism [17]. The activation of defence mechanisms requires specialized receptors, collectively referred to as pattern recognition receptors, such as Toll-like receptors (TLRs) and NOD-like receptors (NLRs) [16,19,20]. Activation of both receptors induces nuclear transcription factor-kappa B (NF-κB), a signalling pathway that regulates immune functions and gene expression related with inflammation and metabolism [21,22,23,24]. Moreover, failure of these regulatory pathways is a trait shared by several intestinal inflammatory diseases, which is also implicated in the development of obesity- and diabetes-related metabolic dysfunction [5].

Mice fed high-saturated fat showed changes in insulin sensitivity and adipose tissue inflammation, associated with the upregulation of *TLR4* and *TLR2* signalling [25]. In intestinal tissue, high-fat diets seem to specifically increase *TLR4* expression [24], but there is less information about the impact of obesogenic diets on the intestinal *TLR2* expression. Throughout the intestinal epithelium, the TLRs are expressed in both immune and non-immune cells, including in enteric neurons, reinforcing their crucial role in intestinal homeostasis [16,20,26,27,28]. In addition, intestinal *TLR2* and *TLR4* expression shows region-specific patterns [21,26,29]. Furthermore, it was shown that high-fat feeding [30] and an intestinal inflammatory state induced the upregulation of intestinal inducible nitric oxide synthase (iNOS) and, hence, NO production, suggesting it to be a crucial immunomodulator of the intestinal motility [31].

In the context of the immune cell sensors involved in metabolic inflammation, there is increasing interest in the role of the NLR family, specifically the *NLRP3* inflammasome [32]. High-fat feeding of NLRP3-deficient mice exhibited a favourable effect on insulin sensitivity and in adipose tissue inflammation related with obesity [32,33]. Moreover, *NLRP3* is induced not only by microbial components, TLR ligands, and the transcription factor NF-κB, but also by fatty acids [22,32,34,35], suggesting its potential regulatory role in metabolic inflammation. However, the contribution of the *NLRP3* inflammasome for the intestinal inflammatory response in diet-induced obese mice models has yet to be elucidated.

Western diets (WDs), typically with low dietary fibre, can shape microbiota composition [14,36,37]. In turn, mucin glycans are used as nutrient sources for bacteria, resulting in an erosion of the mucus barrier, an increase in intestinal permeability, and a decrease in beneficial microbiota-derived metabolites, such as short-chain fatty acids (SCFAs) [18,36,38]. Furthermore, the mucus layer overlaying the epithelium is produced by goblet cells under a direct immune regulation, responding to variable stimuli in a region-dependent manner [39]. Although not fully explored, current evidence has established a causative association between high saturated-fat intake and perturbation of gut microbiota, leading to intestinal permeability, proinflammatory responses, and dysmotility [12,23,27,40,41,42,43]. For example, in mice models, high-fat feeding induced an upregulation of jejunal inflammatory gene transcripts associated with impaired glucose homeostasis, without changing adiposity [40]. Meanwhile, high-fat plus high-sugar feeding induced a higher body weight gain, accompanied by hyperproliferation of intestinal stem cells and changes of enterocytes in a regional-specific manner [44]. Furthermore, distinct effects of a high-fat diet on the intestinal barrier function were also shown in other studies, ranging from marginal in the ileum and colon to severe in other segments of the small intestine [42,45].

It has been shown that intake of added sugars early in life affected intestinal microbiota in rats, and this effect was obesity-independent [46]. Meanwhile, mice on a high-sugar diet exhibited glucose homeostasis impairment and obesity [47], disruption of gut epithelium integrity, and systemic inflammatory response [48]. However, research on the effects of early high-sugar diets on intestinal inflammatory response and its metabolic outcomes is limited.

Whether immune regulation is distinctly affected by the consumption of WD and high-sugar diet (HSD) is still unknown. Therefore, the main goal of this study was to investigate how early unhealthy dietary patterns influence intestinal inflammatory responses and their association with metabolic outcomes in later life, by comparing two specific diet patterns in the development of obesity and its impact on intestinal inflammation.

## 2. Results

### 2.1. Effects of Energy and Macronutrient Intake on Anthropometric Parameters

**Figure 1:**
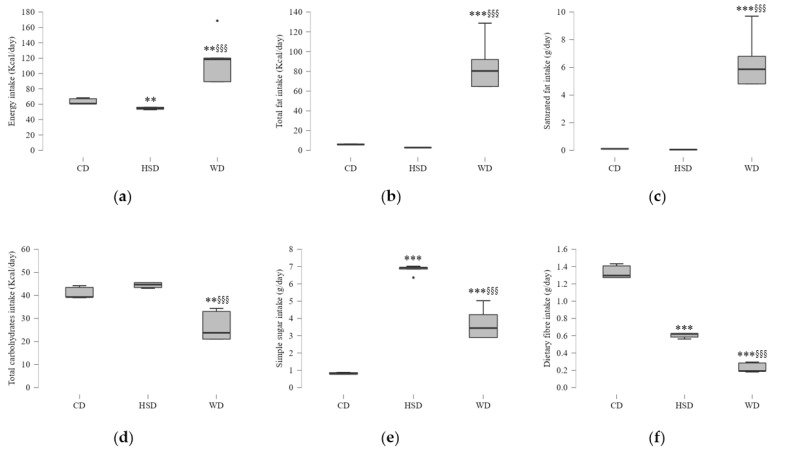
Graphic representation of the daily intake of energy and nutrients from chow-fed (CD), high-sugar diet (HSD), and Western diet (WD) groups. (**a**) Total energy intake from solid food and drink, although CD rats had access to water. (**b**) Energy from total fat intake. (**c**) Saturated fat intake (g/day). (**d**) Energy from total carbohydrate intake (**e**). Simple sugar intake (g/day). (**f**) Dietary fibre intake (g/day). Mean values ± standard deviation (SD) are plotted. Symbols * and ^§^ indicate significant differences (one-way ANOVA) compared to CD-fed group: ** *p* < 0.01, *** *p* < 0.001; HSD-fed group: ^§§§^ *p* < 0.001 (*n* = 9 rats per group).

Data analysis confirmed possible obesogenic effects induced by HSD and WD (details in Section 4) in rats under these dietary constraints from an early age until adulthood. Total energy intake per day consumed by WD-fed rats was higher than the one of rats fed with a chow diet (CD) or HSD (Figure 1a). Regarding diet composition, WD-fed rats had higher energy from fat, particularly high saturated fat intake, and consumed a lower content of dietary fibres compared to the other feeding groups (Figure 1b,c,f). The amount of simple sugar intake in WD-fed rats was higher only when compared to the CD group (Figure 1e). Although HSD-fed rats had free access to sucrose solution instead of water, like CD-fed rats, this group presented a lower total energy intake, caused by a reduction in chow diet intake, which was concomitant with a reduced cellular nutrient supply, including fibre intake (Figure 1d–f). Moreover, protein intake was greater in CD-fed rats than in those fed with a HSD or WD (Supplemental Table S1).

Lifelong dietary intervention induced significant variation on body weight, visceral adipose tissue weight, and adiposity index, while there was no significant difference in body weight gain (Supplemental Figure S1). The mean body weight of WD-fed rats was about 5% and 13% higher than that of CD- and HSD-fed rats, respectively. Nonetheless, WD feeding induced a sharp increase in adipose tissue weight and rats displayed a greater adiposity index compared with HSD- and CD-fed rats (Supplemental Figure S1c,d). Interestingly, HSD-fed rats had the lowest body weight values, but their adipose tissue was about 44% heavier than that of CD-fed rats and 33% lighter than that of WD-fed rats.

**Figure 2:**
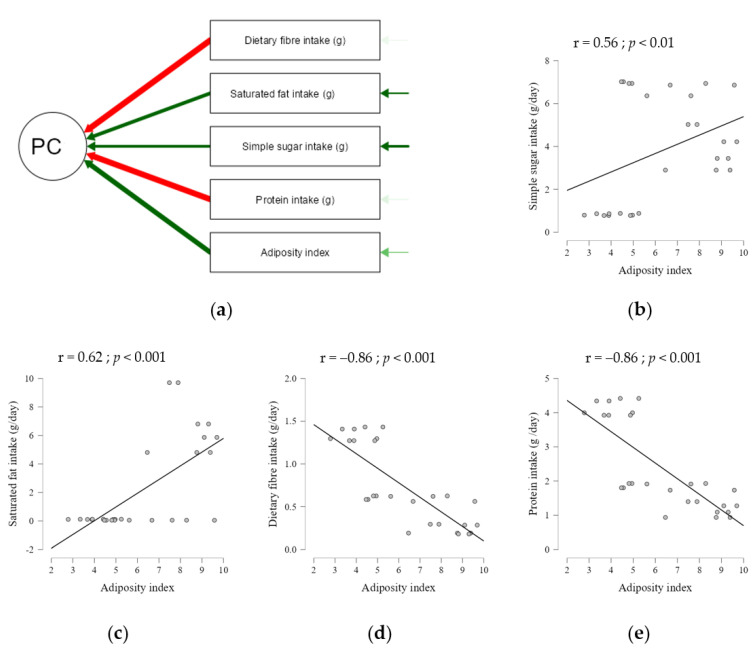
Correlation between nutrients intake and adiposity index. (**a**) Path diagram of principal component (PC) used in Pearson correlation analysis. (**b**) Simple sugar and (**c**) saturated fat intakes were positively correlated with adiposity. (**d**) Dietary fibre and (**e**) protein intakes were negatively correlated with adiposity.

To further investigate the effects of macronutrients on body composition, we performed a Pearson correlation analysis and explored the association between the intake of each dietary component and obesity index (Figure 2), conditioned on body weight gain. We found that all analysed dietary components (Supplemental Table S2) were correlated with the adiposity index. Among these, we showed that saturated fat (g) and sugar (g) intakes were positively correlated with the adiposity index, whereas protein and dietary fibre (g) intakes had a negative correlation (Figure 2d,e).

### 2.2. Dietary Composition Effects on Serum Metabolic Parameters

**Figure 3:**
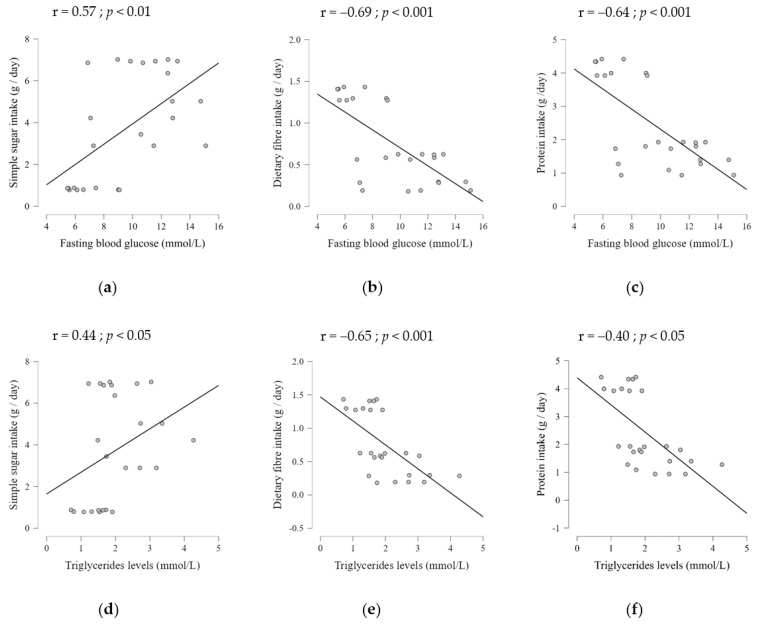
Correlation between nutrients intake and serum metabolic parameters. (**a**) Simple sugar intake had a positive correlation with fasting blood glucose, while (**b**) dietary fibre and (**c**) protein intakes had a negative correlation. (**d**) Simple sugar intake has positively correlated with triglycerides levels. A negative correlation was found between (**e**) dietary fibre and (**f**) protein intakes with triglycerides serum levels.

After showing that both dietary patterns were obesogenic, data analysis sought to evaluate the association between nutrient intake and serum metabolic parameters, namely fasting blood glucose, triglycerides, and total cholesterol levels (expressed as mmol/L). There was a significant difference in the circulating levels of fasting blood glucose between the three groups (Supplemental Figure S2a). Glucose concentration was significantly higher in HSD-fed rats (10.76 ± 2.11) and WD-fed rats (11.48 ± 3.05), compared to the CD feeding group (6.75 ± 1.44; *p* < 0.01). When glucose levels were compared between the HSD and WD feeding groups, we found no significant differences in serum levels. Moreover, Pearson correlation results (Supplemental Table S3) showed that only sugar intake had a positive correlation with fasting blood glucose (Figure 3a–c).

Regarding the blood lipid profile, there was a significant effect of diet on triglyceride and total cholesterol serum levels (Supplemental Figure S2b,c). No significant differences were found in blood lipid profile from rats fed with a WD or HSD, but these values were significantly different when compared with CD-fed rats. In line with the increased adiposity index, triglyceride and cholesterol levels were higher in WD-fed rats than in CD-fed rats (*p* < 0.001). HSD-fed rats had higher levels of triglycerides and total cholesterol than CD-fed rats (*p* = 0.03 and *p* < 0.01, respectively).

Pearson analysis showed that five dietary components were correlated with blood lipid parameters (Supplemental Table S3). Total cholesterol levels were correlated (negatively) only with complex carbohydrate intake, similar to the association found for triglyceride levels. In addition, we found that protein and fibre intake were negatively correlated with triglyceride levels, whereas the mean fat energy, saturated fat, and sugar intakes had a positive correlation (Figure 3d–f, Supplemental Table S3). Furthermore, all serum metabolic parameters were also positively correlated with adiposity (Supplemental Figure S3).

### 2.3. Effects of Obesogenic Diets on the Small Intestine Morphology

**Figure 4:**
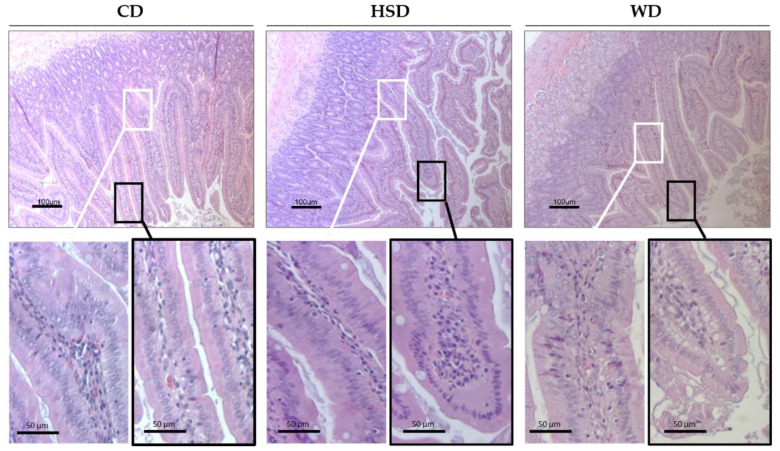
Dietary effects on the small intestine. Representative images of HE-stained duodenum of rats fed with chow diet (CD), high-sugar diet (HSD), and Western diet (WD). The inset shows details of the villi in small intestines, from near the villi base (white box) to the tip (black box). Villi in duodenum from CD-fed rats showed regularly aligned simple columnar epithelium and occasional intraepithelial lymphocytes. HE-staining of tissue sections from HSD-fed rats exhibited enterocytes with misaligned nuclei and slight enlargement of the villus tip. The duodenal villi of HFD-fed rats revealed vacuoles in simple columnar epithelial cells.

Rats fed with a HSD and WD had higher sucrose intake, which is quickly digested into glucose and fructose in the small intestine lumen. To assess whether these dietary patterns can induce changes on intestinal morphology, observation of the duodenal mucosa was undertaken in HE-stained tissue sections (Figure 4). CD-fed rats showed slender duodenal villi with regularly aligned epithelium, mainly composed of enterocytes interspersed with goblet cells and some scattered intraepithelial lymphocytes. Of note, dietary intervention induced changes in duodenal villi characteristics (Figure 4). HSD-fed rats exhibited a slight enlargement of the villi and enterocytes with misaligned nuclei, while vacuoles in the absorptive enterocytes were observed in the small intestine of WD-fed rats. From the tip to villi base, we observed fewer intraepithelial lymphocytes compared to CD feeding.

### 2.4. Distinct Effects of Obesogenic Diets on the Number of Goblet Cells

**Figure 5:**
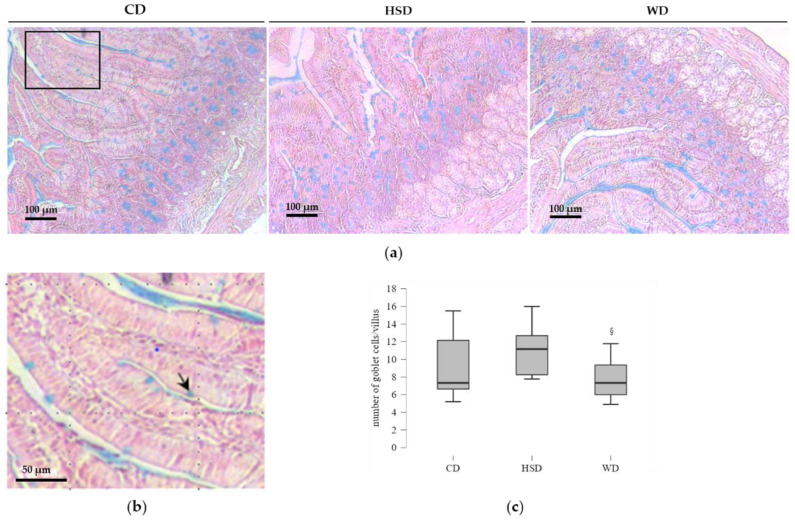
Dietary effects on mucus-secreting cells in the small intestinal epithelium. (**a**) Representative images of Alcian blue staining of transversal sections of duodenum from chow diet (CD), high-sugar diet (HSD), and Western diet (WD) groups. (**b**). Inset of the box showing blue stained goblet cells, which is indicated by an arrow. (**c**) Number of goblet cells per villus of duodenum (*n* = 5–8 villi per rat). Symbol indicates significant differences (one-way ANOVA) compared to HSD-fed group: ^§^ *p* < 0.05.

Next, we examined the HSD and WD feeding effects on the mucus-secreting cells in the same intestine segment. The acidic goblet cells that stained positively with Alcian blue in the villi epithelium were counted (Figure 5).

No significant difference was observed in the goblet cell number between rats fed with a HSD or CD, nor between the WD and CD feeding groups (Figure 5c). Remarkably, the mucus-positive cell number per villi in rats fed with HSD was significantly higher than that in WD-fed rats.

### 2.5. Distinct Effects of Obesogenic Diets on Inflammatory Signalling Pathways in the Small Intestine

Considering that the upregulation of *NF-κB* through *TLR*-dependent signalling triggers the production of proinflammatory cytokines, leading to the impairment of immune functions and chronic low-grade inflammation [16,19,22], we analysed the long-term effects of WD and HSD on the expression of *TLR2*/*TLR4*/*NF-κB* pathways. In addition, the expression of other key mediators for the maintenance of intestinal homeostasis, such as *iNOS* and *NLRP3* [32,34,35,49,50], were analysed.

**Figure 6:**
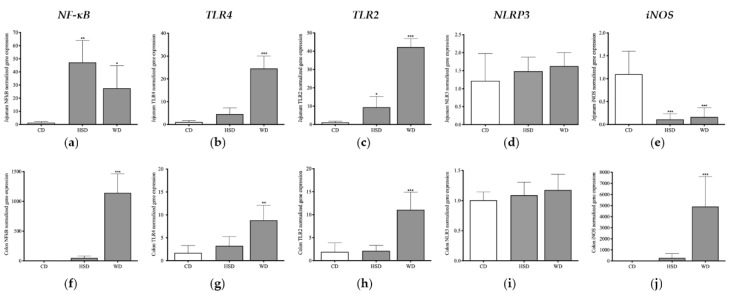
Graphic representation of the normalized expression levels of chow-fed diet (CD), high-sugar diet (HSD), and Western diet (WD), concerning *NF-kB*, *TLR4*, *TLR2*, *NLRP3*, and *iNOS* genes in jejunum (**a**–**e**) and colon (**f**–**j**) samples. Expression levels were normalized against the respective 18S rRNA gene expression and then against the respective controls (CD), set as 1. Mean values ± standard deviation (SD) are plotted. *** *p* < 0.001, ** *p* < 0.01, * *p* < 0.05.

In the jejunum, ANOVA results showed that diets induced significant variation on *NF-κB*, *TLR2*, *TLR4*, and *iNOS* gene expression, but there was no difference in *NLRP3* expression (Figure 6a–e). The small intestine of HSD-fed rats exhibited significantly higher expression of *NF-κB* and *TLR2* compared to CD-fed rats (Figure 6a,c). Although there was a slight increase in the expression of *TLR4* with HSD feeding, the difference was not statistically significant compared to the levels of CD-fed rats (Figure 6b), suggesting that a HSD induced an inflammatory response in the small intestine mainly through the upregulation of the TLR2/NF-κB pathway. Surprisingly, in the jejunum of HSD-fed rats, a reduction in *iNOS* expression, compared to CD-fed rats, was detected (Figure 6c,e). Regarding the WD’s effects on inflammatory gene expression, the upregulation of *NF-κB*, *TLR2*, and *TLR4* expression was also detected in the jejunum compared to CD feeding. In addition, *iNOS* expression levels were reduced in this small intestine segment (Figure 6e).

### 2.6. Distinct Effects of Obesogenic Diets on Inflammatory Signalling Pathways in the Large Intestine

There is evidence linking dietary fats and fibre intake with colonic microbiota composition and inflammation [23]. In this way, we further analysed the diet effects on colonic immune response. ANOVA results showed that dietary intervention also had a significant effect on *NF-κB*, *TLR2*, *TLR4*, and *iNOS* expression in the large intestine, but not on *NLRP3* expression (Figure 6f–j). Notably, there were no significant changes in the colonic expression of *NF-κB*/*TLR2* of HSD-fed rats compared to CD (Figure 6f), neither in *TLR4* expression nor in *iNOS* expression (Figure 6f–j). Meanwhile, WD intake had a significant impact on the activation of colonic-inflammation-associated pathways compared to CD intake. In addition, the upregulation of *NF-κB* expression was higher in the colon, compared with the levels detected in the jejunum (*p* < 0.01). Of note, WD intake induced a significant increase in colonic *iNOS* expression compared to CD intake and with levels in the jejunum (*p* < 0.05), where it was downregulated. 

## 3. Discussion

Overnutrition is recognized as a main driver of the chronic metabolic inflammation associated with the development of obesity and related metabolic disorders [3,5,8,51,52]. There is the concept of dietary fat perturbation of homeostatic microbiota–host interaction, leading to intestinal immune dysregulation and barrier disruption [10,27], enabling bacterial translocation and, consequently, metabolic inflammation [13,43,53]. Both short-term and prolonged exposure to diets with high saturated fatty acids were found to induce inflammatory responses in the intestine and insulin resistance through a *TLR4*/*NF-κB*-dependent mechanism [24,54]. Other studies proposed a fatty acid-specific effect on the intestinal inflammation, through the activation of both the *TLR2* and *TLR4* signalling pathways, leading to low-grade inflammation and glucose homeostasis impairment [13,15,55].

Our results reveal that, in rats submitted to the WD, the *TLR2*/*TLR4*/*NF-κB* signalling cascade activation induced an inflammatory response in the small intestine, with a more exacerbated expression in the colon. Moreover, the colonic inflammatory response to WD was accompanied by an increased expression of *iNOS*, although its expression levels were decreased in the small intestine. In addition, we showed that high intake of saturated fat and added sugar intake by young rats were correlated with worse metabolic profiles in adulthood. It was previously reported that, in mice, diet more severely affected the intestinal barrier in the proximal small intestine than in the colon [27,42,45]. However, other previous findings showed diet-induced colonic inflammation and intestinal permeability occurring through the induction of the *TLR4* and *iNOS* signalling pathways [54]. In addition, dietary fat induces disruption of colonic microbiota and triggers bacterial growth in the small intestine in mice [56]. Together, these findings suggest that early consumption of a WD induces disruption of regulatory signalling pathways in a region-specific manner, providing support to the causative link between ultra-processed food intake, colonic inflammation, and metabolic dysfunction.

Interestingly, we found that HSD feeding induced the activation of the *TLR2*/*NF-κB* pathway only in the small intestine. In line with previous studies, we observed morphological alterations in the small intestine induced by high sugar intake, including a higher number of goblet cells compared with WD feeding. The crucial role played by the *TLR2*-mediated signalling pathways in the regulation of the intestinal epithelial barrier has been recognized [16,29]. *TLR2* expression is shaped by the microbiota, and it reflects bacterial load, displaying low expression levels in the small intestine and higher ones in the proximal colon [26]. In the small intestine, microbiota shaping associated with the upregulation of *TLR2* expression leads to an increased cell turnover [21], changes in tight junction protein expression, and stimulation of mucus secretion by goblet cells [16,19]. In addition, early HSD feeding triggered an increase in adiposity in adulthood, which was correlated with unfavourable metabolic parameters. Indeed, previous studies reported the negative impact of high sugar intake on intestinal structure and function [52,57], as well as on other metabolic organs [48,58]. Therefore, it is plausible to suggest that a sustained high luminal concentration of glucose due to high sugar, per se, induces the upregulation of *TLR2* expression in the small intestine and, consequently, may lead to chronic intestinal permeability, hyperglycaemia [52,57] and, metabolic endotoxemia [8], which may contribute to the onset and progression of obesity.

We showed that only WD feeding induced the *NF-κB*/*TLR4*/*iNOS* signalling activation in the colon. Since both obesogenic diets were low-fibre, it is plausible to suggest that the deregulation of *iNOS* signalling pathway induced by saturated fat might be an internal signal to the disruption of microbiota–host interactions, leading to colonic inflammatory response and dysmotility. Increasing evidence supports the link between diet-induced obesity and intestinal motility disorders, indicating dietary fat as a main contributor to dysfunction of myenteric neurons, which leads to intestinal motility disorders [20,23,31]. Diet-induced colonic inflammation and dysmotility were reported to involve the activation of *TLR4* and *iNOS* signalling [20,23,31]. Dietary fibre intake strongly influences the microbiota–host interactions, interfering with overall metabolic function and, locally, modulating intestinal homeostasis [14,18,23,36,37]. This non-digestible carbohydrate is used by the bacteria mainly in the large intestine, producing SCFA and indole derivatives, which modulate several intestinal functions, such as barrier integrity, energy homeostasis, gut hormone production, and motility [14,23,37].

Convincing evidence links *iNOS* levels with microbiota-immune-nerve interactions, indicating the deregulation of *iNOS* signalling to play a role in colonic dysmotility [31,59]. A concept that emerged from animal models of colitis [31,59,60] linked colonic inflammation with the expansion of *Proteobacteria* [59] and increased oxidative stress [31,59], triggering the upregulation of *iNOS* expression in enteric glial and immune cells [31,60,61]. In addition, dietary fat was shown to induce colonic inflammation through the activation of *NF-κB*/*TLR4* signalling, leading to an increase in *iNOS* expression [23] and a subsequent increase in NO levels released by enteric glia cells in an excitation state [60]. Moreover, our previous study showed that consumption of ultra-processed foods induced the expansion of *Proteobacteria* [62], supporting the hypothesis that the diet-related *iNOS* expression in colon can be caused by disruption of microbiota-immune-nerve crosstalk, contributing to colonic dysfunction [31,60].

We found a downregulation of jejunal *iNOS* expression in rats fed with ultra-processed foods and sugar drinks. Dietary fat was previously reported to induce an increase in *iNOS* expression in the small intestine from rats [30] and mice [40]. This divergence may be explained, at least in part, by differences in animal strains and the nutrient composition used, not only in dietary intervention timing and duration, but also in the small intestine segment analysed. Nevertheless, in *iNOS*-deficient mice, a sustained increase in the bacterial load in the ileum was shown, suggesting that *iNOS* expression might be important for inhibition of a bacterial reflux from the large intestine [49]. Since we found a decrease in *iNOS* expression in the small intestine due to WD and HSD feeding, it is possible that this effect could be the consequence of a chronic defect in the immune response, caused, in part, by dietary effects on the jejunal bacterial load. In addition, we observed fewer intraepithelial lymphocytes in the duodenal tissue sections of WD-fed rats, corroborating previous studies [63]. Alongside with *iNOS* and NO roles in intestinal barrier function [49], their role in the regulation of enteric glucagon-like peptide-1 (GLP-1) sensitivity through a *CD14*/*TLR4* dependent mechanism is also proposed [64]. Indeed, the expression of GLP-1 receptors was shown to be reduced in the jejunum of mice on a high fat diet but was unchanged in the colon [64]. It can be hypothesized that a sustained intestinal proinflammatory responses triggered by early dietary patterns affects immune system development, leading to intestinal function impair and deregulation of gut hormone secretion as well as causing a low-grade systemic inflammation and subsequent infiltration of immune cells in several metabolic organs [8,13,23,40,41,42,44].

During acute inflammatory response, the small and large intestines showed an upregulation of *NLRP3* expression upon injury or microbial stimuli, involving the activation of *TLR4* and *TLR2* signalling [34,35]. Besides the activation by bacterial infection, this signalling pathway can be activated by saturated fatty acids [32,35], being suggested to play a role in immune dysfunction and chronic inflammation, triggering diet-induced obesity and insulin resistance [32,33]. Interestingly, we found that neither WD nor HSD feeding had influence on *NLRP3* expression in the small and large intestines. These results provide information suggesting that saturated fat intake induced neither a direct effect on the activation of intestinal *NLRP3* nor an indirect effect through the bacteria, although we cannot exclude the possibility of *NLRP3* inflammasome activation by other stimuli. The existence of an acute inflammatory response induced by high saturated fat is not expected, though a low-grade inflammation is expected. Indeed, contrarily to other metabolic tissues, the intestine possesses a tissue-resident phagocyte population that is hyporesponsive to microbial stimulation [34], suggesting that inflammasome activation might have tolerance towards non-pathogenic microbiota.

Taken together, the data indicate that the chronic consumption of ultra-processed foods induces exuberant colonic inflammatory response, contributing to metabolic consequences in adulthood, whereas the effects of sugar beverages on inflammation mechanisms were mainly observed in the small intestine. These observations support the concept that the intestinal structure and its responsive mechanisms can be disrupted by direct and indirect effects of different nutrients in a regional-specific manner.

## 4. Material and Methods

### 4.1. Animals and Experimental Diets

All procedures in this study were reviewed and approved by the Animal Care and Use Committee (ORBEA) of the Faculty of Medicine of the University of Porto (Portugal). The procedures were performed by accredited scientists (licensed users of the Federation of Laboratory Animal Science Associations—FELASA) and conducted according to the European Union Directive (2010/63/EU) on the protection of animals for scientific purposes. Data in the current paper pertain to rats involved in a dietary manipulation experiment, some aspects of which have already been published. These previous publications have included patterns of body weight, adiposity, serum lipid profile, and serum glucose levels [65].

We previously exposed male Wistar rats, aged 4 weeks, to three dietary treatments (*n* = 9 per diet group) for 14 weeks. The number of animals per group considered previous studies in our lab and the fact that HSD group presented a statistically significant difference at “*p*” values, ranging from 0.05–0.02 and at varying 80–90% probability (levels of power) for *n* = 9 and the WD group, where the number need was lower. Throughout the study, animals had ad libitum access to liquid and solid food, which was replaced daily. The control diet group (CD) was fed with standard chow diet (4RF21/C Mucedola, Settimo Milanese, Italy) and had access to water. The high-sugar diet group (HSD) was fed with standard chow diet and had access to 30% sucrose solution (Sigma-Aldrich Company Ltd., Madrid, Spain; 1.2 Kcal/mL). The Western diet group (WD) was fed with standard chow diet mixed with a selection of palatable human foods and had access to 15% sucrose solution (0.6 Kcal/mL). Full details on macronutrient composition of the diets are found in the supplementary material (Table S1). During the whole experimental period, rats were housed two per cage to avoid social isolation, under controlled 22–24 °C temperature and 12:12 h light/dark cycle conditions. More information about procedures and experimental design can be found in our previous paper [65]. Food intake was obtained by subtracting the final weight of the food from its initial weight, including any food pellets that had spilled into the cage.

### 4.2. Tissue Collection

Animals were sacrificed under deep anaesthesia, induced with sevoflurane (SevoFlo, Abbott Laboratories Ltd., Maidenhead, UK). The small and large intestines were dissected and washed in PBS to remove luminal contents. Then, ~2 cm-long sections of the duodenum were collected and fixed in 4% (*w*/*v*) neutral-buffered formalin solution (Sigma-Aldrich) for 24 h at room temperature. Tissue samples of jejunum and colon were quickly frozen and stored at −80 °C, until being processed for extraction of total RNA for quantitative real-time PCR analysis. Visceral adipose tissue (VAT) was weighted to assess the degree of obesity, by using the adiposity index, expressed as the ratio of VAT weight to body weight (g/100 g).

### 4.3. Histological Analysis and Goblet Cell Staining

Specimens of cross-section segments of duodenum were embedded in low-melt paraffin wax and cut into 4 μm thick histological sections on a microtome (Shandon^TM^ Finesse^TM^ 325, Thermo Scientific, Runcorn, UK). For histopathological examination, tissue sections were stained with hematoxylin and eosin (HE) (Diapath, Martinengo, Italy) and evaluated according to: widening and shortening of intestinal villi, infiltration of intraepithelial lymphocytes, and nucleus position within the absorptive cells (enterocytes) of the intestinal epithelium. The HE sections were selected for quantitative evaluation when the duodenal villi were well-oriented, displaying a surface epithelium composed mainly of enterocytes intermixed with goblet cells. Away from the regenerative zone of villus, the enterocytes were identified as columnar cells with an oval nucleus, normally occupying a uniformly basal position in the enterocyte. The differentiation between enterocytes and goblet cells was recognized by the typical pear shape of goblet cells. The Alcian blue staining (at pH 2.5) counterstained with hematoxylin was used to quantify goblet cells that contained acid non-sulphated mucins. Goblet cells were counted in both sides of the villi from base towards the tip of the villi, excluding the stem cell zone. Only villi that were cut longitudinally and with defined limits were used for quantification of goblet cells. The number of goblet cells in villi per tissue section was counted, and the average number of goblet cells per villus was estimated on 5–8 villi per animal (*n* = 9/group). This histological assessment was performed in a blind manner to the group by one of the authors. Slides were observed under phase contrast microscopy, using 100× and 600× magnifications (Eclipse TE2000-U microscope, Nikon, Melville, NY, USA), coupled to a DXM1200F digital camera and controlled by Nikon ACT-1 software (version 2.70).

### 4.4. Gene Expression Analysis

At the time of harvest, total RNA was extracted from jejunum and mid colon samples using the TripleXtractor reagent (Grisp, Porto, Portugal), in accordance with the instructions of the manufacturer. In total, 1 μg of the total RNA of each sample was reversely transcribed into cDNA using the GRS cDNA Synthesis Mastermix (Grisp, Porto, Portugal), in a total volume of 20 μL. cDNA templates were then amplified with specific primers for target genes, *iNOS*, *NF-Κb*, *NLRP3*, *TLR2* and *TLR4*, using Xpert Fast SYBR Mastermix (Grisp, Porto, Portugal) in a C1000 Touch^TM^ thermocycler, equipped with a CFX96 Touch Real-Time Detection System (Bio-Rad Laboratories, Hercules, CA, USA) and analysed with the CFX Maestro^TM^ software, version 2.3 (Bio-Rad Laboratories), in accordance with the protocol of the manufacturer.

Product amplification was performed with 1 μL of sample cDNA, corresponding to 50 ng of DNA, analysed in duplicate in a final volume of 20 μL/well, containing 10 μM of each primer (iNOS forward primer: 5′-CCAGAGCCTCATCGGTCGTC-3′; *iNOS* reverse primer: 5′-GGGTCCTTCCGCAGACAAC-3′; *NF-κB* forward primer: 5′-CCAGAGCCTCATCGGTCGTC-3′; *NF-κB* reverse primer: 5′-GGGTCCTTCCGCAGACAAC-3′; *NLRP3* forward primer: 5′-CCAGAGCCTCATCGGTCGTC-3′; *NLRP3* reverse primer: 5′-GGGTCCTTCCGCAGACAAC-3′; *TLR2* forward primer: 5′-CCAGAGCCTCATCGGTCGTC-3′; *TLR2* reverse primer: 5′-GGGTCCTTCCGCAGACAAC-3′; *TLR4* forward primer: 5′-CCAGAGCCTCATCGGTCGTC-3′; *TLR4* reverse primer: 5′-GGGTCCTTCCGCAGACAAC-3′). The PCR program run was as follows: (a) denaturation at 95 °C, 3 min; (b) amplification in 55 cycles (denaturation at 94 °C, 20 s; annealing at 55 °C, 30 s, and extension at 72 °C, for 30 s), followed by plate reads; (c) melt curve plotting between 65 °C and 95 °C, with 0.5 °C increments every 5 s, followed by plate reads. The change in gene expression was calculated using the 2^−^^Δ(ΔCt)^ comparative method with the housekeeping gene 18S ribosomal RNA (18S rRNA) as the internal gene (18S rRNA forward primer: 5′-TTCGGAACTGAGGCCATGATT-3′; 18S rRNA reverse primer: 5′-TTTCGCTCTGGTCCGTCTTG-3′), though the presented data were calculated by using the 18S rRNA gene normalized to the control group.

### 4.5. Correlation Analysis with Physiological Traits

Body VAT weight, adiposity index, and serum parameters in the end of dietary intervention were correlated (Pearson’s correlation) with average daily intake of macronutrients.

### 4.6. Statistical Analysis

Data were tested for normal distribution and homogeneity of variances (Shapiro–Wilk test) and adjusted Welch test, when appropriate. The effects of treatment (HSD and WD) on all other variables were assessed by one-way analysis of variance (ANOVA). Pairwise comparisons between diet groups were determined either by the post hoc Tukey’s test or the non-parametric Kruskal–Wallis test, followed by Dunn’s multiple comparisons test. For correlation analysis, the Pearson correlation coefficients were used, with a confidence interval of 95%. The statistical analysis was performed using JASP open-source software (JASP, version 0.16.2.0, University of Amsterdam, Amsterdam, The Netherlands). A *p* < 0.05 was considered as statistically significant.

## 5. Conclusions

This study demonstrated that WD upregulates the *TLR2*/*TLR4*/*NF-κB* pathway axis and modulates intestinal *iNOS* expression, enhancing inflammatory response in the colon. The consumption of simple sugar activated a jejunal inflammatory response through the TLR2/NF-κB signalling pathway.

## Data Availability

The data that support the findings of this study are available from the corresponding author upon reasonable request.

## Supplementary Materials

The following supporting information can be downloaded at: https://www.mdpi.com/article/10.3390/ijms231810984/s1.
