# Supplementary material for: Unhealthy Diets Induce Distinct and Regional Effects on Intestinal Inflammatory Signalling Pathways and Long-Lasting Metabolic Dysfunction in Rats
Source: Int J Mol Sci. 2022 Sep 19;23(18):10984. doi: 10.3390/ijms231810984 (PMC9503261; doi:10.3390/ijms231810984)
Supplement: Supplementary file 1 [file ijms-23-10984-s001.zip › ijms-1866408-supplementary.pdf]

Supplementary Materials for

Unhealthy diets induce distinct and regional effects on intestinal inflammatory signalling pathways and long-lasting metabolic dysfunction in rats

Nogueira *et al*

Corresponding author: brunofonseca@ff.up.pt

Includes: Tables 1 to 3; Figs 1 to 3

Table S1. Statistics of energy and nutrient intake in rats fed with chow (CD), high-sugar (HSD) and Western (WD) diets.

**Daily intake**	**CD**		**HSD**		**WD**		***p*-value**		
	Mean	Std. Deviation	Mean	Std. Deviation	Mean	Std. Deviation	CD vs HSD	CD vs WD	HSD vs WD
Energy intake (Kcal)	63.86	3.71	54.45	1.34	120.29	30.74	3.74×10^{-4}	3.74×10^{-4}	3.74×10^{-4}
Protein energy intake (%)	25.95	0.19	13.62	0.48	3.90	0.42	3.53×10^{-4}	3.53×10^{-4}	3.74×10^{-4}
Total carbohydrate energy intake (%)	64.58	0.26	81.42	0.65	22.96	3.22	3.74×10^{-4}	3.74×10^{-4}	3.74×10^{-4}
Carbohydrate intake (Kcal)	37.95	2.3	17.09	0.79	12.12	3.00	3.74×10^{-4}	3.74×10^{-4}	1.36×10^{-3}
Simple sugar intake (Kcal)	3.30	0.18	27.24	1.04	15.14	3.48	3.74×10^{-4}	3.74×10^{-4}	3.74×10^{-4}
Total fat energy intake (%)	9.47	0.07	4.96	0.17	73.14	3.58	3.36×10^{-4}	3.36×10^{-4}	3.74×10^{-4}

Unsaturated fat intake (Kcal)	5.03	0.28	2.25	0.10	29.27	7.86	3.74×10^{-4}	3.74×10^{-4}	3.74×10^{-4}
Saturated fat intake (Kcal)	1.01	0.06	0.45	0.02	59.16	17.44	3.74×10^{-4}	3.74×10^{-4}	3.74×10^{-4}

Note: *p*-value of Mann-Whitney U test. *n* = 9 per group.

Table S2. Pearson's partial correlations analysis between adiposity index and nutrient intake.

**Parameter**	**Daily intake**	**Pearson's r**	***p*-value**	Significant correlations, one-tailed
Adiposity index	Simple sugar intake (g)	0.56**	1.57×10^{-3}	** $p < 0.01$
	Total fat energy intake (%)	0.64***	2.08×10^{-4}	*** $p < 0.001$
	Unsaturated fat intake (g)	0.57**	1.18×10^{-3}	** $p < 0.01$
	Saturated fat intake (g)	0.62***	3.80×10^{-4}	*** $p < 0.001$
	Energy intake (Kcal)	0.48**	6.20×10^{-3}	** $p < 0.01$
	Protein energy intake (%)	-0.85***	1.53×10^{-8}	*** $p < 0.001$
	Total carbohydrate energy intake (%)	-0.49**	5.40×10^{-3}	** $p < 0.01$
	Carbohydrate intake (g)	-0.86***	1.11×10^{-8}	*** $p < 0.001$
	Dietary fibre intake (g)	-0.86***	6.21×10^{-9}	*** $p < 0.001$

Note: Conditioned on variables: Body weight grain. * $p < 0.05$, ** $p < 0.01$, *** $p < 0.001$, one-tailed

Table S3. Pearson's partial correlation analysis between serum biochemical levels and nutrient intake.

**Serum parameter**	**Daily intake**	**Pearson's r**	***p*-value**	Significant correlations, one-tailed
Fasting blood glucose (mmol/L)	Simple sugar intake (g)	0.49**	7.16×10^{-3}	** $p < 0.01$
	Total fat energy intake (%)	0.20	0.17	Ns
	Unsaturated fat intake (g)	0.24	0.13	Ns
	Saturated fat intake (g)	0.31	0.07	Ns
	Energy intake (Kcal)	0.24	0.87	Ns
	Protein energy intake (%)	-0.63***	4.63×10^{-4}	*** $p < 0.001$
	Total carbohydrate energy intake (%)	-0.05	0.40	Ns
	Carbohydrate intake (g)	-0.63***	4.30×10^{-4}	*** $p < 0.001$
	Dietary fibre intake (g)	-0.64***	3.54×10^{-4}	*** $p < 0.001$
Triglyceride serum levels (mmol/L)	Simple sugar intake (g)	0.18	0.20	Ns
	Total fat energy intake (%)	0.34*	0.05	* $p < 0.05$
	Unsaturated fat intake (g)	0.34	0.05	Ns
	Saturated fat intake (g)	0.38*	0.04	* $p < 0.05$
	Energy intake (Kcal)	0.32	0.06	Ns
	Total carbohydrate energy intake (%)	-0.25	0.12	Ns
	Protein energy intake (%)	-0.49**	7.24×10^{-3}	** $p < 0.01$
	Carbohydrate intake (g)	-0.35*	0.04	* $p < 0.05$
	Dietary fibre intake (g)	-0.43*	0.02	* $p < 0.05$

Total cholesterol levels (mmol/L)	Simple sugar intake (g)	0.21	0.34	Ns
	Total fat energy intake (%)	0.03	0.88	Ns
	Unsaturated fat intake (g)	-0.11	0.62	Ns
	Saturated fat intake (g)	-0.06	0.77	Ns
	Energy intake (Kcal)	-0.17	0.22	Ns
	Total carbohydrate energy intake (%)	0.04	0.43	Ns
	Protein energy intake (%)	-0.28	0.10	Ns
	Carbohydrate intake (g)	-0.38*	0.04	* $p < 0.05$
	Dietary fibre intake (g)	-0.33	0.06	Ns

Note: Conditioned on variables: Adiposity index. * $p < 0.05$, ** $p < 0.01$, *** $p < 0.001$, one-tailed

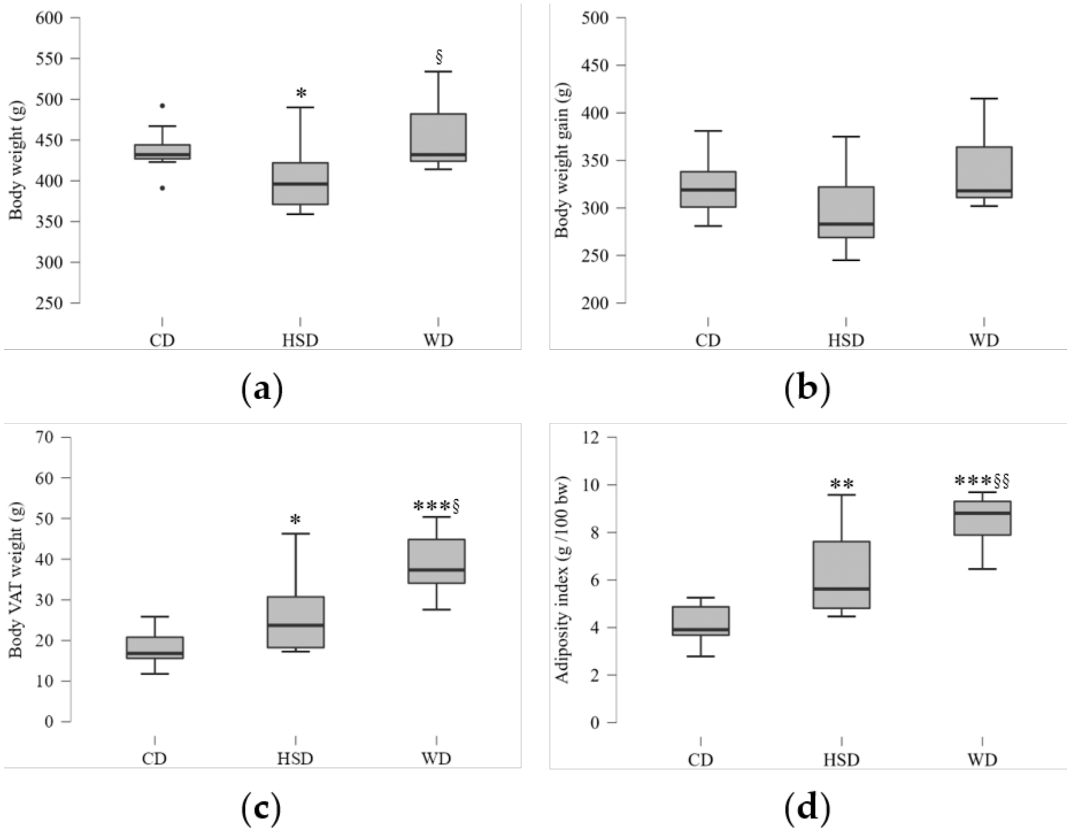

Figure S1. Effects of dietary intervention on body weight, adipose tissue weight and adiposity**.** (**a**) Body weight after 14 weeks of feeding. (**b**) Total body weight gain. (**c**) Total visceral adipose tissue (VAT) weight. (**d**) Adiposity index determined as total VAT per 100 g of body weight (bw). Mean values ± SD are plotted. Symbols indicate significant differences (one-way ANOVA) compared to CD-fed group: * $p < 0.05$, ** $p < 0.01$, *** $p < 0.001$ and HSD-fed group: § $p < 0.05$, §§ $p < 0.01$ ($n = 9$ rats per group). *p*-values reported for Welch statistic and using Dunn's test for multiple comparisons within each variable. CD, chow diet, HSD, high-sugar diet; WD, western diet.

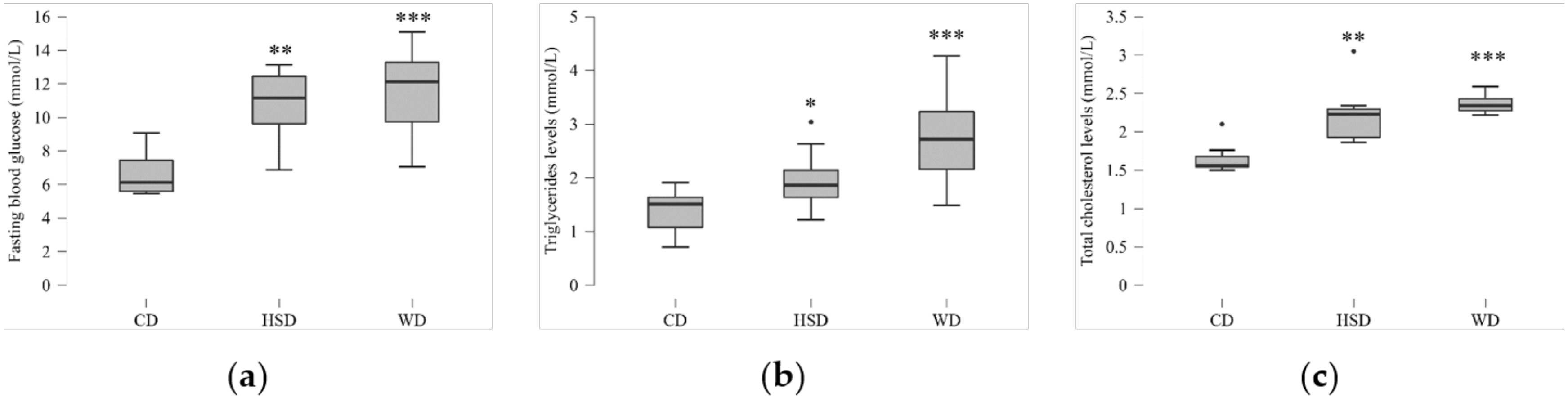

Figure S2. Effects of dietary intervention on serum metabolic parameters of rats fed with chow (CD), high-sugar (HSD) and Western (WD) diets. Serum levels of fasting blood glucose (**a**), triglycerides (**b**) and total cholesterol (**c**) were significantly higher in both HSD and WD-fed rats. Mean values ± SD are plotted. Symbol indicate significant differences (one-way ANOVA) compared to CD-fed group: * $p < 0.05$, ** $p < 0.01$, *** $p < 0.001$, ($n = 9$ rats per group). p-values reported for Welch statistic and using Dunn's test for multiple comparisons within each variable.

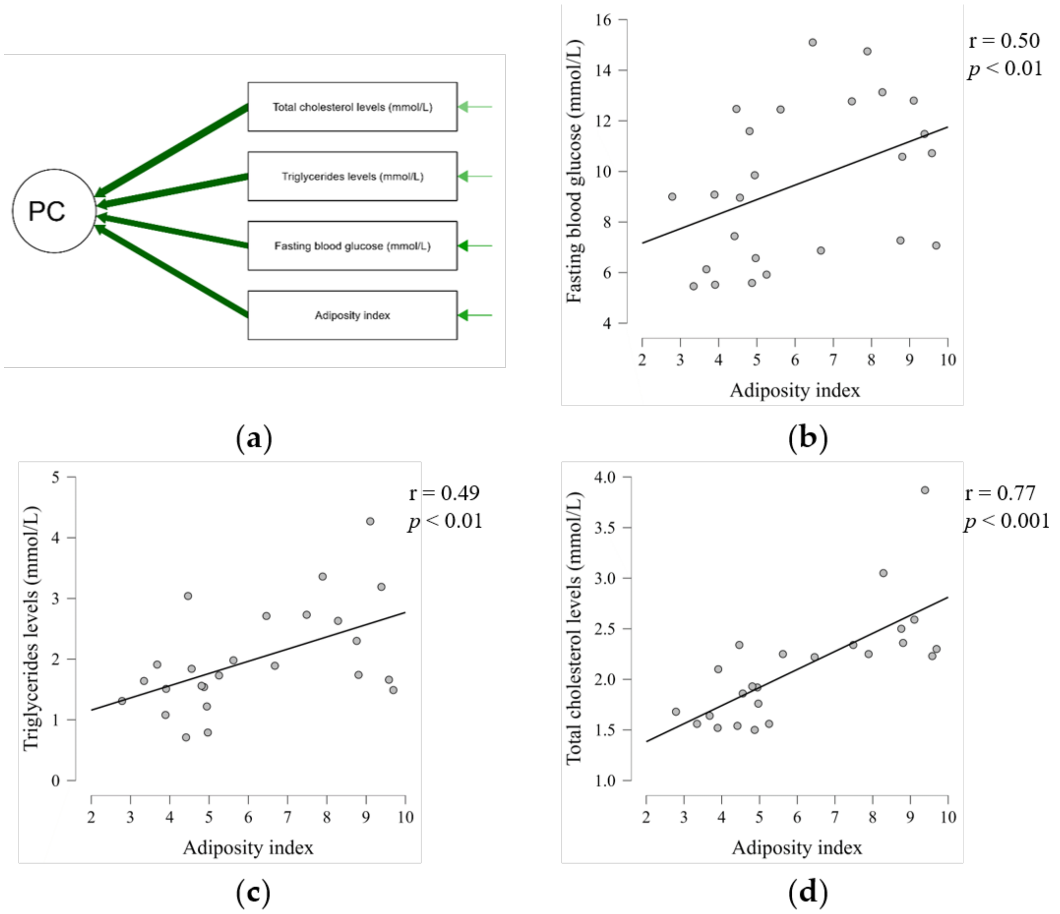

Figure S3. Correlation between serum metabolic parameters and adiposity index. (**a**) Path diagram of principal component (PC) used in Pearson correlation analysis. (**b**) Fasting blood glucose, (**c**) triglycerides and (**d**) total cholesterol levels correlated positively with adiposity.
